# Spontaneous Hemorrhage into the Pseudocyst of the Pancreas Without Pseudoaneurysm: A Report of Rare Case and Literature Review

**DOI:** 10.7759/cureus.68151

**Published:** 2024-08-29

**Authors:** Satyanarayana Kummari, Sairam Subburam, Rithika Ramadugu, Pushpahaas Jamalapuram, Mahipal Rangi

**Affiliations:** 1 Radiology, All India Institute of Medical Sciences, Nagpur, IND; 2 General Practice, Government Medical College, Omandurar Government Estate, Chennai, IND; 3 General Practice, Kamineni Academy of Medical Sciences, Hyderabad, IND; 4 Radiology, MNR Medical College, Sangareddy, IND

**Keywords:** acute pancreatitis (ap), chronic pancreatitis (cp), acute on chronic pancreatitis, open cystogastrostomy, digital subtraction angiography(dsa), computed tomography (ct ), pancreatic pseudocyst hemorrhage, pancreatic pseudocyst (ppc)

## Abstract

The pseudocysts of the pancreas usually occur in cases of acute or chronic pancreatitis due to damage to the pancreatic ducts. Alcohol abuse is the most common cause of acute or chronic pancreatitis. Hemorrhage into the pseudocyst is one of the most lethal complications of pancreatic pseudocyst. In this article, we present the case of a 49-year-old male patient who presented to the emergency room with primary symptoms of pain in the upper abdomen and vomiting that had been occurring for two days and had worsened over the past eight hours. He is a follow-up case of chronic pancreatitis, as well as stable pseudocysts located in the lesser sac, peripancreatic, and epigastric regions. Additionally, the patient had a history of alcohol misuse. The contrast-enhanced computed tomography (CECT) examination of the abdomen and pelvis revealed an enlarged pancreas, hypodense and heterogeneously enhancing pancreatic parenchyma, diffuse peripancreatic fat stranding, and fluid collections. There are a few well-defined hypodense, peripherally enhancing lesions in the lesser sac, peripancreatic, and epigastric regions. On a plain computed tomography (CT) scan, the lesion in the lesser sac showed hyperdense (65 HU) and heterogeneous areas, indicating intracystic hemorrhage. On CT angiography and digital subtraction angiography (DSA), there was no detectable source of bleeding into the pseudocyst. The patient was diagnosed with acute-on-chronic pancreatitis with pseudocysts and spontaneous hemorrhage in the pseudocyst without the presence of a pseudoaneurysm. Conservative treatment was recommended as the patient was hemodynamically stable, and no pseudoaneurysms were detected on the CECT or DSA. The patient exhibited a positive response to the treatment and was discharged in stable condition. The patient was recommended to have a conclusive procedure at a later date. A cystogastrostomy was performed after a period of one month. The postoperative recovery was unremarkable. The purpose of this case report is to highlight the significance of using computed tomography (CT) and angiography for promptly identifying the rare occurrence of hemorrhage into the pseudocyst of the pancreas. Additionally, it emphasized the uncommon occurrence of hemorrhage in the pseudocysts, along with their typical presentation and radiological evaluation.

## Introduction

Pancreatic pseudocysts are non-epithelial lined collections that form as a result of pancreatic duct injury, typically caused by alcoholism or biliary stones. These cysts are common in cases of chronic pancreatitis but can also be observed in cases of acute pancreatitis or pancreatic trauma [1,2]. The potential complications of the pseudocyst of the pancreas include the compression of the gastric outlet, bile duct, duodenum, and major abdominal vessels, as well as infection and bleeding into the pseudocyst [3,4]. Spontaneous intracystic hemorrhage, also known as hemorrhagic pseudocyst of the pancreas, is a rare complication in acute or chronic pancreatitis, occurring in about 6% to 17% of cases. However, it is a life-threatening condition with a mortality rate of approximately 40% [5]. Here, we present a 49-year-old male patient with acute on chronic pancreatitis with multiple pseudocysts and spontaneous hemorrhage in the pseudocyst without the presence of a pseudoaneurysm.

## Case presentation

The 49-year-old male patient presented at the emergency room with primary symptoms of pain in the upper abdomen and vomiting that had been occurring for two days and had worsened over the past eight hours. Positive tenderness and rebound pain were present in the upper abdomen, which was otherwise flat. He is a follow-up case of chronic pancreatitis, as well as stable pseudocysts located in the lesser sac, peripancreatic, and epigastric regions. Additionally, the patient had a history of alcohol misuse and seizures. The following were the findings from the lab: The white blood cell count was 15,430 cells per cumm, the hemoglobin level was 10.5 g/dL, the amylase level was 398 units per liter, the lipase level was 626.0 units per liter, the serum calcium level was 2.36 millimoles per liter, and the glucose level was 7.6 millimoles per liter. The lactate dehydrogenase level was 177 units per liter. The amount of C-reactive protein was 141 mg/L. Prothrombin time: 11.2 seconds; activated partial thromboplastin time: 36.3 seconds.

**Figure 1:**
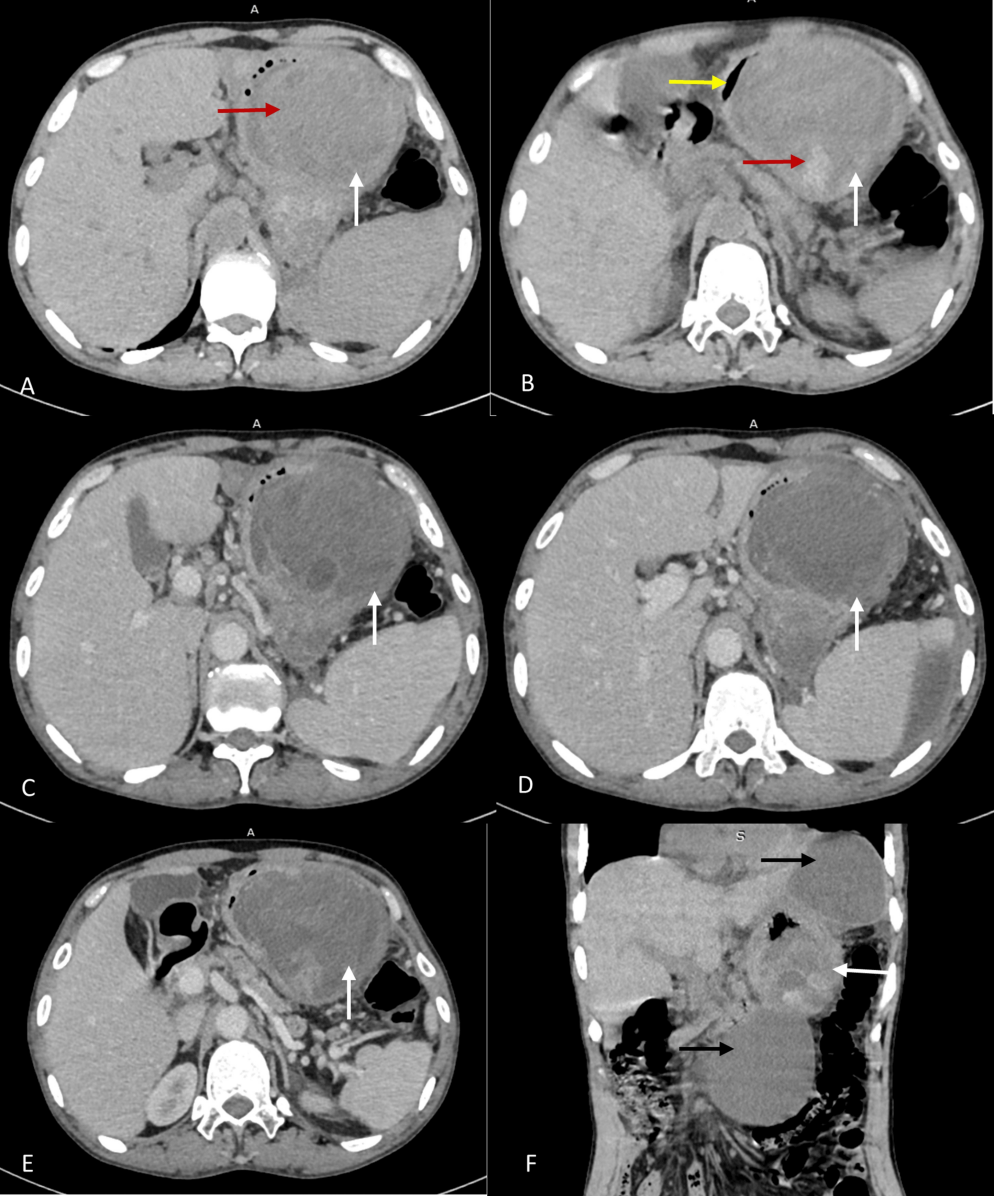
NCCT and CECT of abdomen and pelvis. (A) and (B): NCCT axial views; (C)-(E): CECT axial views images show well-defined hypodense lesion (pseudocyst-white arrows) with hyperdense and heterogeneous areas (hemorrhage-red arrows) in the lesser sac causing significant mass effect on stomach (yellow arrow). (F) NCCT coronal view: image shows three well-defined hypodense lesions in the lesser sac and epigastric region (uncomplicated pseudocysts-black arrows and hemorrhagic pseudocyst-white arrow). The lesion in the lesser sac with hyperdense and heterogeneous areas. NCCT: non-contrast computed tomography, CECT: contrast-enhanced computed tomography.

The ultrasound of the abdomen and pelvis revealed an enlarged pancreas with a marked decrease in echogenicity, surrounding fat stranding, and fluid collections. A few well-defined hypoechoic lesions of varying sizes were noted in the lesser sac, peripancreatic, and epigastric regions. The lesion in the lesser sac showed hyperechoic areas and internal echoes, indicating intracystic hemorrhage. The contrast-enhanced computed tomography (CECT) examination of the abdomen and pelvis revealed an enlarged pancreas, hypodense and heterogeneously enhancing pancreatic parenchyma, diffuse peripancreatic fat stranding, and fluid collections. Few well-defined hypodense, peripherally enhancing lesions of varying sizes were noted in the lesser sac, peripancreatic, and epigastric regions. On a plain scan, the lesion in the lesser sac showed hyperdense (65 HU) and heterogeneous areas, indicating intracystic hemorrhage. The lesions exhibited a significant mass effect on adjacent structures. On computed tomography (CT) angiography and digital subtraction angiography (DSA), there was no detectable source of bleeding into the pseudocyst (Figure 1). 

Based on the medical history of a patient and the findings from additional investigations, the patient was diagnosed with acute on chronic pancreatitis with pseudocysts and spontaneous hemorrhage in the pseudocyst without the presence of a pseudoaneurysm. Hemorrhage into the pseudocyst may be caused by the erosion of adjacent capillaries or venules that are close to the pseudocyst. Conservative treatment was recommended as the patient was hemodynamically stable and no pseudoaneurysms were detected on the CECT or DSA. The patient exhibited a positive response to the treatment and was discharged in stable condition. The patient was recommended to have a conclusive procedure at a later date. A cystogastrostomy was performed after a period of one month. The postoperative recovery was unremarkable; there was no additional indication of bleeding, and the hemoglobin level remained constant. After a period of three weeks, he was able to consume a regular diet without any issues and was eventually discharged from the hospital.

## Discussion

Pancreatic pseudocysts are non-epithelial lined collections that form in and around the pancreas as a result of pancreatic duct injury. The incidence of pancreatic pseudocysts is estimated to be between 1.6 and 4.5 cases per 100,000 adults annually, making up around two-thirds of all pancreatic cystic lesions [1]. It occurs due to injury to the pancreatic ducts, usually due to alcoholism or biliary stones. The occurrence has been reported to be higher among acute on chronic pancreatitis (41.8%) as compared to acute pancreatitis (14.6%). Most pancreatic pseudocysts resolve spontaneously. Clinically, most of the cases are asymptomatic, and the symptomatic patients present with chronic abdominal pain, gastric outlet obstruction, obstructive jaundice, or symptoms of chronic pancreatitis [2].

The potential complications of the pseudocyst of the pancreas include the compression of the gastric outlet, bile duct, duodenum, and major abdominal vessels, rupture of the pseudocyst, as well as infection and bleeding into the pseudocyst [3,4]. Spontaneous intracystic hemorrhage, also known as hemorrhagic pseudocyst of the pancreas, is a rare complication in acute or chronic pancreatitis, occurring in about 6% to 17% of cases. However, it is a life-threatening condition with a mortality rate of approximately 40%. A hemorrhagic pseudocyst can manifest as recurring abdominal pain, tenderness in the abdomen, anemia, upper or lower GI bleeding, and a pulsating palpable mass in the abdomen [5].

The rupture may occur due to a continuous process of pancreatic tissue necrosis and exudation, leading to a gradual accumulation of pathogenic elements, excessive internal pressure, and ultimately causing the rupture. There is also the possibility that the cyst or its contents could erode the blood vessels, which would result in the vessels rupturing and bleeding. Furthermore, cystic compression or vascular embolism may cause portal hypertension, which results in the rupture and bleeding of portal-systemic collaterals or varices. Additionally, if the abdomen is subjected to external force, it might result in the rupture of cystic lesions [6]. The pseudocyst exerts direct pressure on the blood vessels, leading to erosion into the visceral cavity and auto-digestion of the artery wall by proteolytic enzymes present in the pseudocyst or during a severe inflammatory episode of acute pancreatitis. Commonly involved blood vessels are the splenic artery (30-50%), gastroduodenal artery (17%), and pancreaticoduodenal artery (11%) [4]. Formation of fistula connecting to duodenum, common bile duct, esophagus or colon may be seen, presenting with upper gastrointestinal or lower gastrointestinal bleed. Hemorrhage occurs in only 2.5% of all pancreatic pseudocysts. The hemorrhagic pseudocyst might rupture into the retroperitoneum, causing retroperitoneal hemorrhage; into the peritoneum, causing hemosuccus pancreatitis or intraperitoneal hemorrhage; biliary tract, causing hemobilia; or into the ampulla of the vater, causing gastrointestinal hemorrhage [7,8]. 

A patient who has a history of chronic pancreatitis or pancreatic pseudocyst should be suspected of having hemorrhage in the pseudocyst if there is any sudden decrease in hematocrit, sudden enlargement of the pseudocyst, and no evidence of upper gastrointestinal bleeding or upper GI bleeding with no other source of bleeding seen on the upper gastrointestinal endoscopy. Clinically, an auscultation for bruit must be done. An esophagogastroduodenoscopy must be done for confirmation [9].

Radiological diagnosis is made by ultrasonography with color Doppler, CECT abdomen, MRI-magnetic resonance cholangiopancreatography (MRCP), and angiography. The CECT of the abdomen and pelvis is the preferred imaging technique, with a high sensitivity ranging from 82% to 100% and a specificity of approximately 98%. The conclusive diagnosis is established by identifying the connection between the pseudocyst and the feeding artery [10]. In this particular case, on both the CT angiogram and the DSA, there was no clearly identifiable source of bleeding into the pseudocyst. A possible source of bleeding into the pseudocyst could be the erosion of capillaries or venules that are close to the pseudocyst.

The spontaneous resolution of pseudocyst is commonly seen, particularly in cases that arise following an episode of acute pancreatitis. In the majority of cases involving uncomplicated pseudocysts, conservative treatment is recommended. However, pseudocysts that arise as a consequence of chronic pancreatitis seldom resolve spontaneously. Angiographic embolization and selective percutaneous arterial embolization are the two potential treatment options for hemorrhagic pseudocysts resulting from pseudoaneurysm. In situations where embolization is unsuccessful, persons who are unable to undergo embolization, or cases where endoscopic therapy is not successful, a surgical approach may be considered. However, it is generally not the preferred option due to mortality rates of 20-29% in hemodynamically unstable cases. There are three primary types of invasive procedures used to treat pseudocysts: percutaneous drainage, endoscopic drainage, and surgical drainage or excision. The surgical procedure entails the drainage of the pseudocyst into the gastrointestinal tract, along with the partial removal of the pancreas and spleen [4,9,10].

## Conclusions

Imaging should be performed to confirm the presence or absence of pancreatic pseudocysts in patients with acute or chronic pancreatitis. If pancreatic pseudocysts are confirmed, ongoing investigations should be performed to monitor their progression; the absence of regression or any increase in size necessitates further evaluation. The diagnosis of hemorrhage in a pseudocyst is frequently delayed due to its uncommon occurrence and varied clinical manifestations. In patients with a pseudocyst or a history of chronic pancreatitis, it is important to maintain a high level of suspicion when they present with acute abdominal pain, a sudden decrease in hematocrit, sudden enlargement of the pseudocyst without any signs of upper gastrointestinal bleeding, and upper gastrointestinal bleeding without any other detectable source of bleeding on upper gastrointestinal endoscopy. We recommend using both CECT and angiographic evaluation to promptly diagnose hemorrhage in the pancreatic pseudocyst, thereby facilitating the planning of relevant therapeutic strategies.
